# Cell Sorting-Directed Selection of Bacterial Cells in Bigger Sizes Analyzed by Imaging Flow Cytometry during Experimental Evolution

**DOI:** 10.3390/ijms24043243

**Published:** 2023-02-07

**Authors:** Di Tian, Caiyan Wang, Yunfei Liu, Yueyue Zhang, Adriano Caliari, Hui Lu, Yang Xia, Boying Xu, Jian Xu, Tetsuya Yomo

**Affiliations:** Laboratory of Biology and Information Science, School of Life Sciences, East China Normal University, Shanghai 200062, China

**Keywords:** bacterial morphology, cell sorting, experimental evolution, imaging flow cytometry

## Abstract

Cell morphology is an essential and phenotypic trait that can be easily tracked during adaptation and evolution to environmental changes. Thanks to the rapid development of quantitative analytical techniques for large populations of cells based on their optical properties, morphology can be easily determined and tracked during experimental evolution. Furthermore, the directed evolution of new culturable morphological phenotypes can find use in synthetic biology to refine fermentation processes. It remains unknown whether and how fast we can obtain a stable mutant with distinct morphologies using fluorescence-activated cell sorting (FACS)-directed experimental evolution. Taking advantage of FACS and imaging flow cytometry (IFC), we direct the experimental evolution of the *E. coli* population undergoing continuous passage of sorted cells with specific optical properties. After ten rounds of sorting and culturing, a lineage with large cells resulting from incomplete closure of the division ring was obtained. Genome sequencing highlighted a stop-gain mutation in *amiC*, leading to a dysfunctional AmiC division protein. The combination of FACS-based selection with IFC analysis to track the evolution of the bacteria population in real-time holds promise to rapidly select and culture new morphologies and association tendencies with many potential applications.

## 1. Introduction

Bacteria exhibit large morphological variability associated with each species, strain and growth condition. This diversity includes rod, coccoid, spirilla types, and more uncommon shapes such as bean, tapered, coryneform, ovoid, star and dendroid as a result of various selective pressures [1,2,3]. Environmental factors can alter it in response to internal and external fluctuations, such as cell division and segregation, gene mutation, antibiotics (e.g., β-lactams), and nutrient access [2]. Given its fundamental impact on nutrient diffusion and many other fundamental properties, bacterial morphology is tightly controlled genetically as a determinant of cell identity [4,5]. Cell elongation and division are usually driven by the cytoskeletal protein FtsZ, MreB, and their related protein complex [6,7,8]. These structural proteins couple cell growth and division with the cell wall, and the overall cell shape is cemented in place by cell wall synthesis [5]. Mutations associated with these cytoskeleton complexes often result in changes in the cell length and width [9,10,11,12,13,14], which still require further explorations to further understand the gene-morphology relationship in detail.

Bacterial cell morphology is also a trait subject to selective pressures and thus morphological variation is an adaptation avenue to external environmental changes. Upon resource starvations, *Bacillus subtilis* can form smaller coccoid spores via sporulation [1,15] and *Escherichia coli* changes shape from rod to filament [16]. The same phenomenon can be also seen in *Proteus mirabilis,* which shifts from the short rod into the hyperflagellated and elongated status to avoid capture during the transition to swarming motility [17,18]. *Salmonella* cells double the average length of swarmer cells and the number of flagella per cell during swarming, resulting in a significant increase in motor power [19]. Such morphological plasticity in bacteria indicates that bacterial morphology has a high potential for directed adaption in the laboratory under specific experimental conditions, such as Lenski’s long-term laboratory evolution [20,21,22] and our previous study where *E. coli* was adapted in an oleic acid vesicle (OAV)-rich medium [23].

It is then critical to conduct qualitative and quantitative cell analysis to better understand how bacteria adapt to and evolve in the environment by maintaining and modifying their shape. To date, various advanced technologies, including advanced microscopy and flow cytometry (FCM), have been developed to analyze the dynamics of cell morphology at either the single cell or population level [24]. Over the last few decades, FCM-based platforms such as the fluorescence-activated cell sorter (FACS) and imaging flow cytometer (IFC) have provided a better solution for cell quantification, biomass estimation, and cell sorting in a more sophisticated way [25,26]. Cell parameters such as light scatter values and specific fluorescent channels are used in FCM to analyze the broad heterogeneity of bacterial communities [27,28,29], assess bacterial viability by measuring both live and dead bacteria with fluorescent dyes [24,30], and to monitor bacterial morphological changes in specific conditions [29,31]. Remarkably, a cell shape mutant in *Helicobacter pylori* was successfully isolated through a single round of FACS enrichment based on low forward scatter (FSC) value, leading to the rapid identification of multiple genes affecting its morphology [32]. Similarly, an *E. coli* suppressor mutant was enriched and isolated by FACS, leading to the discovery of a new class of enzymes required to suppress cell shape defects [33]. Furthermore, our previous study also employed FACS as a cell size gate to direct laboratory evolution in *E. coli,* and found that the evolved cells were smaller than the ancestor within 400 generations [34]. FACS is thus a powerful tool for isolating and enriching cells with novel morphological phenotypes during laboratory evolution, which might not be directly associated with a certain gene mutant. The combination of these powerful analytical tools in the directed evolution of bacteria is a promising avenue in the development of synthetic biology approaches to obtain modified strains with desirable properties by providing a way to monitor morphological evolution and to select for it quickly [35]. However, it remains unknown whether we could obtain stable cells with morphological mutants from wild-type bacteria under a defined selection threshold and how many generations and sorting cycles are sufficient during experimental evolutions.

Here, we applied morphological directed evolution by selecting cell subpopulations of larger size via FACS from *E. coli* populations. The dynamics of cell morphologies through sequential passages were also monitored by IFC during laboratory evolutions. After multiple rounds of selection, we found that the bacteria from one of the experimental groups evolved to a larger size and formed long chains. The evolved cells are morphologically different from wild-type *E. coli*, confirmed by FACS, IFC, confocal microscopy, and scanning electron microscopy. Whole genome re-sequencing revealed a stop-gain mutation in *amiC*, a gene related to cell division in the cell population. Collectively, our results demonstrate that bacteria can adapt to directed selection pressure by FACS through accumulating genomic mutation related to cell shapes. These findings may be applied to the directed evolution of morphological mutants more suited to human needs that may be involved in novel approaches to fermentation as designed microbial cell factories in synthetic biology [35].

## 2. Results

### 2.1. Dynamics of Bacterial Morphology in Cell Sorting-Directed Experimental Evolutions

**Figure 1:**
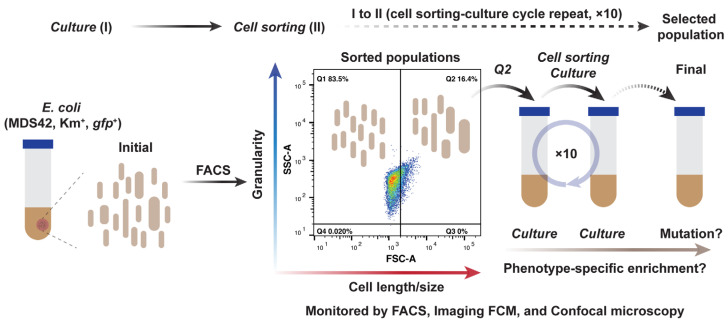
Schematics of strategies for cell sorting-guided cell size selection. It shows the rounds of directed size selection by using FACS. The cells in bigger sizes were defined as the gated cells from Q2 based on the forward scatter value (FSC). The Q2-gated cells were then subjected to sorting-culture cycles 10 times. Parallel experiments with continuous passages without cell sorting were conducted as an internal control. Three replicates from control (Control-A, -B, -C) and sorting-guided (FACS-A, -B, -C) groups were investigated to monitor cell morphology and genomic changes.

To explore the natural evolution of bacteria with potentially larger cell sizes, we employed cell sorting technology to sort the *E. coli* cells based on their optical properties. As illustrated in Figure 1, the laboratory *E*. *coli* strain *MDS42ΔgalK::P_tet_-gfp-kan* (free of mutation-generating IS elements and a reduced evolutionary potential) was used as an ancestor [36]. Cell size and shape were correlated to the FSC value in flow cytometry [37]. Here, we designed a directed selection strategy where a threshold of 2000 (Q2 gate in Figure 1) was fixed to sort the gated bigger *E. coli* cells with a higher FSC value (See Materials and methods for details).

**Figure 2:**
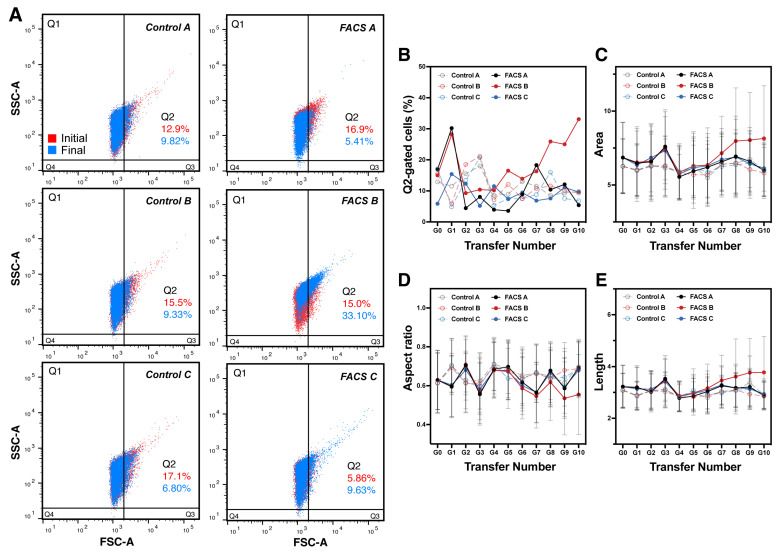
Dynamics of cell morphology of passaged cells with or without cell sorting pressure. (**A**) Cell population analysis via FACS represented initial (G0, red) and final (10th cycles, G10, in blue) cells. The percentages of six gated cell populations were indicated. Detailed dynamics of all 10 cycles (G1–G10) were shown in (**B**) (Population of Q2-gated cells), (**C**) (Cell area), (**D**) (Aspect ratio), and (**E**) (Length). The red line shows the trend of each parameter from the FACS-B group.

During the sorting-directed evolutions of *E. coli* cells, we observed changes in bacterial cell size. After 10 rounds of the sorting-culture cycle, we first compared the percentage of Q2 gated cell populations among cells from the initial (G0) to final (G10) passages in the control and FACS groups, respectively. As shown in Figure 2A, the percentage of Q2 gated cell populations was decreased in all lineages from the control and one lineage of FACS-directed groups, except the FACS-B and FACS-C lineages (Figure 2A). Remarkably, Q2 cell % in the FACS-B group reaches 33.10% of the total cell populations. In addition, the dynamics of Q2-gated cell populations from Figure 2B demonstrate that the level of fluctuations in FACS-directed groups (~5–35%) is more severe than that in control groups (~5–20%), implying that the selection force from FACS works well during the experimental evolution. The dynamic trend further revealed that cells from FACS-B have a significant increase in the Q2-gated cell population starting from G7 while others keep fluctuating within 10 rounds of passages with or without selection from FACS (Figure 2B). However, there was no difference among the FACS-A, FACS-C lineages, and the three control groups, indicating that they might need more rounds of sorting-culture cycles for cells from FACS-A/C lineages to enrich cells with a bigger size such as the one in the FACS-B lineage.

To further understand the dynamics of bacterial morphology during experimental evolutions, several parameters related to cell morphology, such as area (Figure 2C), aspect ratio (Figure 2D), and length (Figure 2E), were analyzed in parallel by IFC during the sorting process for all lineages. As expected, a gradual increase in cell area (Figure 2C, red line) and length (Figure 2E, red line), together with a significant decrease in aspect ratio (Figure 2D, red line), can only be observed for the FACS-B lineage. The decrease in aspect ratio indicates that the cells in the FACS-B group become longer during experimental evolution, which is consistent with the cell length and area value directly observed in IFC. Although the other two FACS-directed lineages showed no remarkable size changes after 10 rounds of selection, longer evolution times may be needed to see dramatic shifts, since there is a clear tendency to larger sizes between the control and FACS groups.

### 2.2. Verifications of Cell Morphologies via Various Microscopic Technologies

**Figure 3:**
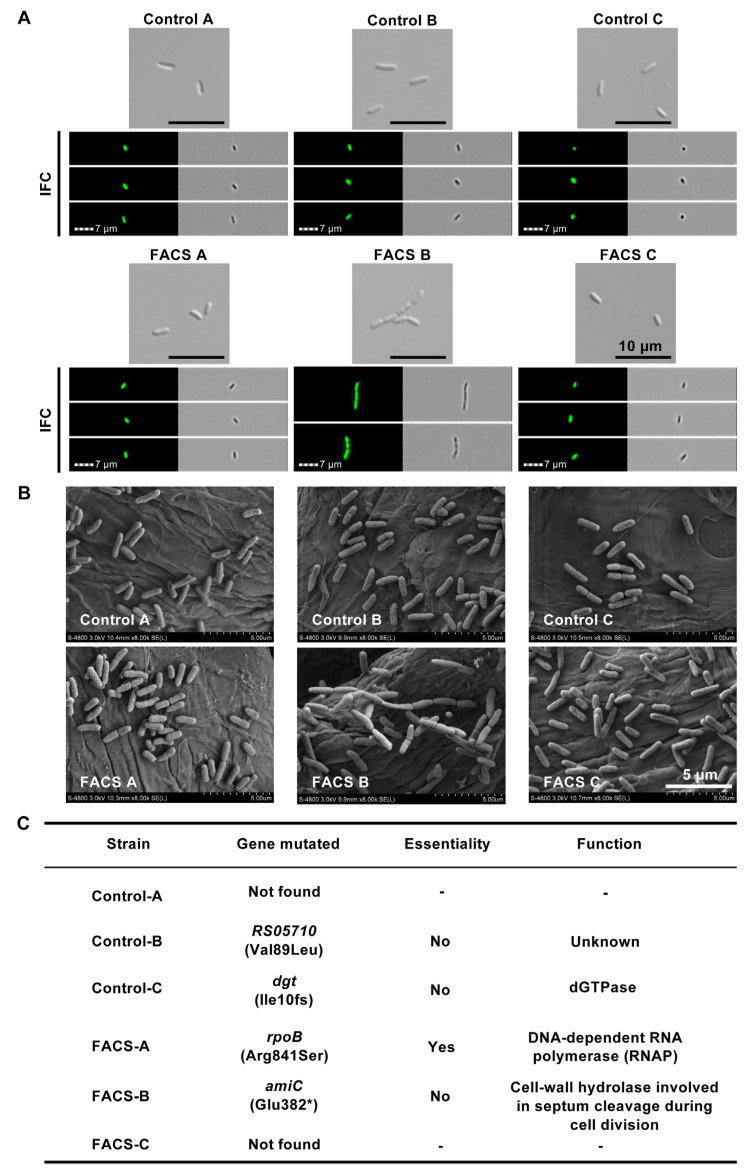
The changes in morphology and genomics through continuous cell sorting and passages. Images of G10 cell groups from optical images (Scale bar = 10 μm), flow cytometry (Scale bar = 7 μm), and scanning electron microscope (SEM, Scale bar = 5 μm) were shown in (**A**) and (**B**), respectively. Cells from sorting-culture group B (FACS B) clearly demonstrated a growth phenotype in long chains of unseparated cells. Genome mutations were spotted by genome re-sequencing, and the specific gene mutations of all passaged strains were listed in (**C**).

Bacteria morphologies observed in FACS and IFC were examined by confocal microscopy to confirm our previous observation because a long filamentous cell phenotype can take one of two forms: either a single, elongated cell with an increased cell volume, or a long chain form of multiple cells with a constant volume [34]. IFC was used to discriminate between the two phenotypes since it provides images for all detected cells (as shown in the lower panel of Figure 3A) and is thus more suited to this task than FACS. The typical cell morphology of the G10 population was also confirmed directly by confocal microscopy (Figure 3, upper panel) and SEM (Figure 3B). We confirmed that the shape of bacteria in the FACS-B group indeed became longer. Unlike what was observed in the FACS-B group, the cell morphology in the FACS-A and FACS-C groups did not change significantly when compared to the control groups (Figure 3A,B). The cells in the control groups and the FACS-A and FACS-C groups maintained a rod shape, whereas the FACS-B group displayed a major population of cells with a growth phenotype in long chains of unseparated cells, indicating that there might be some mutation(s) in the genes involved in the cell division or separation. Collectively, the microscopic results clearly indicate that the FACS and IFC analysis that we successfully selected the evolved *E. coli* cells with a detectable cell size-related phenotype.

### 2.3. Whole Genome Sequencing Revealed a Mutation in the amiC Gene

**Figure 4:**
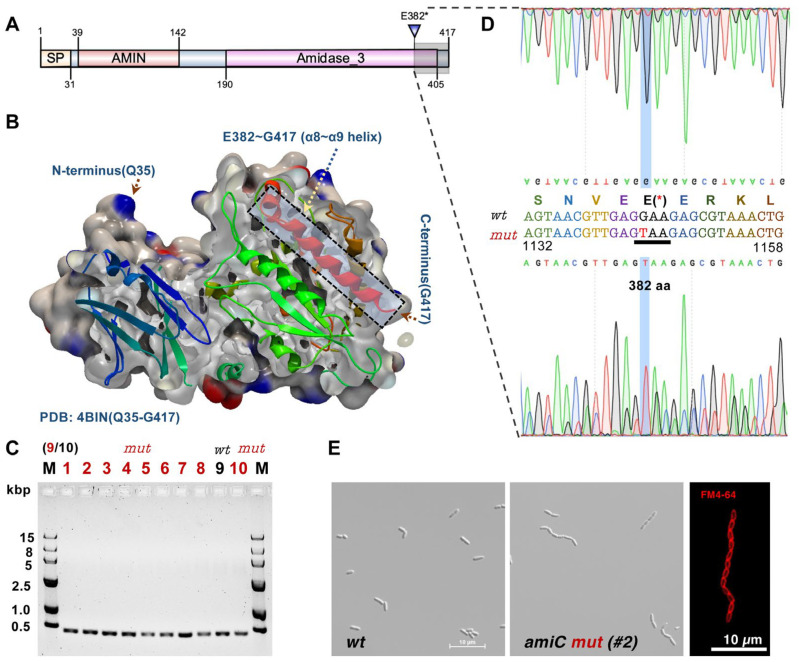
C-terminal deletion (E382*) of AmiC induces cells with a long-chain phenotype. (**A**) The domain information was depicted based on Pfam. SP: signal peptide; AMIN/Amidase_3: typical domains of an amidase. (**B**) The three-dimensional structure of *E. coli* AmiC was adopted from PDB (4BIN) and illustrated in a CLC sequence viewer (8.0). The C-terminal deleted region (α8–α9 helix) from E382 was shown in a transparent box. A further 10 single colonies were screened (**C**) and sequenced (**D**) for the *amiC* gene locus. (**E**) Observation and comparison between the wild-type and mutant cells (left two panels: optical microscopy with bright field; right panel: *amiC* mutant cells stained with FM4-64 membrane dye). Scale bar = 10 μm.

To further determine which gene mutations caused cell division failure in the FACS-B lineage, the whole genome re-sequencing for all six populations was performed. The detailed results are shown in Table S1 and further summarized in Figure 3C. The sequencing showed four mutations: RS05710 (Val89Leu) in Control-B, *dgt* (Ile10fs) in Control-C, *rpoB* (Arg841Ser) in FACS-A, and *amiC* (Glu382*) in FACS-B, while no mutations were found in the Control-A and FACS-C lineages. Although three of the mutations have no direct relationship with cell morphology and cell division, we identified one single-base substitution in the coding region of *N*-acetylmuramyl-l-alanine amidase (*amiC*) which is an essential cell-wall hydrolase involved in septum cleavage during cell division [38]. The nucleotide substitution at position 1144 (G to T) led to an early stop at amino acid (aa) position 382 (E382*) (Figure 3C and Figure 4A), which might cause a deficiency of the AmiC protein as an amidase.

### 2.4. Long-Chain Phenotype Mediated by C-Terminal Loss of the amiC Gene

As illustrated in Figure 4A, AmiC mainly contains two structurally independent domains: the N-terminal AMIN domain and the C-terminal catalytic domain, where the N-terminal AMIN domain is a peptidoglycan-binding domain involved in the localization of AmiC at the division site, whereas the C-terminal catalytic domain comprises multiple alpha helices (α1–α9) which control the activation of AmiC [39]. From the three-dimensional structure of AmiC (PDB: 4BIN), the deletion of C-terminal aa382–417 results in the loss of the α8–α9 helix from the C-terminal catalytic domain (Figure 4B).

To further confirm the *amiC* mutant, we then screened ten random colonies from the plate culture of the FACS-B G10 population for a strain with a pure AmiC Glu382* mutation. Figure 4C shows that 90% (9/10) of the cells hold the same mutation in *amiC* as verified by sequencing the PCR products (Figure 4D). In addition, the cells of the pure amiC* colony were also cultured and validated for long-chain phenotype via confocal microscopy as compared to the wild-type strain (Figure 4E, left and middle panels). We also used a lipophilic dye FM4-64 to stain the inner membrane, and chained cells were connected without separation of the cell membrane, suggesting that cells with long-chain morphology can be accumulated by FACS in our designed cell gate.

## 3. Discussion

Employing the FACS-directed laboratory evolution, we successfully obtained an evolved *E. coli* population (FACS-B) with a size-related phenotype. Previous investigations demonstrated that the degree of change in bacterial morphology is affected by the strength of size selection in the directed evolution experiment [34]. Therefore, we speculated that the results in the FACS-A and FACS-C groups could be attributed to insufficient selection times or the weak strength of the size selection. Another point that needs to be mentioned is the large degree of phenotypic fluctuation in cell length among the isogenic bacterial cells during the cell growth and cell division, such as the rod-shaped *E. coli* cells used in this study [40,41]. In this regard, the selection window might be very narrow in early sorting-culture cycles for efficiently enriching cells with possible morphological changes including size, length, etc. We reported previously that selection also affected phenotypic fluctuation under a single selection environment in *E. coli* [42]. It might be interesting to perform further experimental evolutions by tuning the threshold of the cell gate for cell sorting to see if the success rate and sorting-culture cycles can be improved. In addition, the use of different cell gates with other selective conditions such as antibiotic treatment to monitor the output of the experimental evolution is also of great interest.

We observed that the mutant population rapidly becomes predominant after short-term selection-directed evolution, which is one advantage of this approach where mutants can be identified in a time-effective manner. Another possibility might exist in that a low number of cell passages after cell sorting may cause the accidental fixation of growth defect mutants through genetic drift, although ~1000 cells were subsequently employed for each passage in this study. A previous study revealed that a cell shape was successfully isolated through a single round of enrichment from a double lens-equipped FACS, leading to the rapid identification of multiple genes affecting the morphology in *H*. *pylori* [32]. With experimental evolution and IFC technology, we might be able to investigate the dynamic of enriched bacterial mutants from the angles of evolutionary biology and ecology at either the single cell level and/or the population level. The morphological manipulation of bacteria cells and colonies could have a significant impact on the field of synthetic biology [35], thanks in big part to the rapid development of new high-throughput cell sorting and imaging-based analytical techniques [43].

Peptidoglycan (PG) is a dynamic structure that is constantly modified and remodeled throughout cell growth and division [44]. During cell division, cytoskeletal proteins, PG hydrolases, and associated regulatory proteins governed the synthesis and subsequent hydrolysis of septal PG, thus allowing the separation of the two daughter cells [8,45]. AmiC plays an essential role in the final step of cellular division by cleaving the septal PG to divide one constricted cell into two daughter cells [38,46]. In addition to *E. coli*, the essential role of periplasmic amidase in separating the daughter cells during cell division has been characterized in various bacterial species, such as *Vibrio cholerae* [47], *Neisseria gonorrhoeae* [48], and *Caulobacter crescentus* [49]. There are three periplasmic amidases, AmiA, AmiB, and AmiC in *E*. *coli,* and the absence of one or more periplasmic amidases causes the formation of long chains with uncleaved septa [50,51]. In terms of AmiC, previous functional studies indicated that four strictly conserved residues (His196, His265, Glu211, and Glu373) of the C-terminal catalytic domain might be involved in the catalytic activity of AmiC [52]. Moreover, some non-conserved residues in the α5–α6 helix seem to be necessary for the stabilization of the helix in the active site [39]. In this study, the long-chain cell morphology in the FACS-B group also confirmed the critical role of AmiC in cell division and further suggested that the α8–α9 helix and subsequent (aa382–417) of the C-terminal catalytic domain are also essential for AmiC. However, the role of the α8–α9 helix residual domain in AmiC is still unknown and needs further investigation, for example, by probing its interaction with its activator NlpD [39].

Although our results indicated a relationship between the *amiC* mutant and the chained cell phenotype, further experimental evidence from a rescue assay is required to verify the dysfunction of amiC* (E382*) obtained in this study. In addition, a recent study reported that growth conditions (e.g., carbon sources, cell membrane and osmotic stresses) affected the phenotype of ∆*amiC* in *Burkholderia insecticola* [53]. It also revealed that an acidic environment could stimulate AmiB and AmiC amidase activity in *E. coli*, where the cell number per chain in various genetic backgrounds was decreased at pH 5.2 as compared with the chained cells at pH 6.9 [54]. Since the current experimental evolutions were performed in a neutral condition (pH 7.0), it would be interesting to investigate the cell morphology under different culture conditions for all obtained endpoint *E. coli* populations.

## 4. Materials and Methods

### 4.1. Bacterial Strain and Medium

A genome-reduced *E. coli* strain, *MDS42ΔgalK::P_tet_-gfp-kan*, was constructed previously and employed in this study [55]. The constitutively expressed green fluorescent protein (GFP) is used as a fluorescent marker to be distinguished in FACS and IFC and to determine if there is possible contamination during experimental evolutions. The bacteria were maintained in M63 minimal medium (pH 7.0) comprising 62 mM K_2_HPO_4_, 39 mM KH_2_PO_4_, 15 mM (NH_4_)_2_SO_4_, 0.009 mM FeSO_4_, 0.015 mM thiamine hydrochloride, 0.2 mM MgSO_4_, and 0.2% (11.1 mM) glucose. The medium was sterilized with 250 mL filtration units equipped with 0.22 μm membranes (Millipore, Bedford, MA, USA).

### 4.2. Cell Sorting and Passage

A single colony of the strain was randomly collected from a 1% agar M63 plate supplemented with 50 μg/mL kanamycin. Liquid culture (3 mL, 50 μg/mL kanamycin) and passage in the M63 medium were conducted in 15 mL conical tubes (ThermoFisher, Waltham, MA, USA). The second-round passage (T2, also termed G0), which showed stable growth in the M63 liquid, was then employed as the starting point of the cell sorting experiment. Considering the loose coupling between bacterial morphology and growth rate, the overnight cultures at the stationary phase with a cell density of ~10^9^ cells/mL were subjected to imaging cell cytometry (Amnis™ ImageStream™X, Seattle, WA, USA) and cell sorting (BD FACSMelody, BD Biosciences, La Jolla, CA, USA). Since the resulting value from forward scatter (FSC) indicates the relative cell size of the tested bacteria [37], we then set the threshold of 2000 (Q2 gate in Figure 1) to sort the bigger cells. Those sorted ~1000 cells in 50 µL fresh medium were then used as the inoculation source for the subsequent culture. The continuous sorting-culture cycle was repeated 10 times (G1–G10), in parallel with the control group without cell sorting procedures.

### 4.3. Imaging Flow Cytometry

The *E. coli* cell populations were analyzed using an Amnis™ ImageStream™X imaging flow cytometer installed with INSPIRE acquisition software (Luminex, Austin, TX, USA), as described in our previous study [23]. Green fluorescence was induced with a 200 mW 488 nm laser, and the emission was detected with a 505–560 nm filter in Channel 2. The bright field data were collected in Channel 4, side scatter (SSC) was produced with a 2 mW 785 nm laser, and emissions were collected in channel 6 with a 745–800 nm filter. The images were acquired with 60-fold magnification, a pixel size of 0.09 μm^2^, a low flow rate, and high sensitivity. SpeedBead calibration reagents (400041, Luminex, Austin, TX, USA) were used for daily calibration as internal beads and run concurrently for real-time velocity detection and autofocus. The cell cultures were diluted with a fresh medium 1~100-fold for measurement. Approximately 10,000 cells (data points) were acquired and gated according to the intensity of fluorescence and aspect ratio to exclude the internal beads and the cell culture debris with IDEAS software (v.6.2.183.0, Luminex, Austin, TX, USA). Only the cell images in focus from IFC were used for the statistical analysis [56]. The relative lengths (L) and widths (W) of the cells were represented by the major and minor axis lengths, which were the longest and narrowest dimensions of the cell image, respectively. The cell shape was represented by the aspect ratio (W/L), which indicates the sphericity of the cell in the image. The relative cell size was represented by two features: area and volume. The relative cell area (*A*) was the total pixels of the cell image, which was calculated in IFC software (v.6.2.183.0, Luminex, Austin, TX, USA).

### 4.4. Confocal Microscopy and Scanning Electron Microscopy (SEM)

Live-cell imaging of the bacteria cells cultured was performed in an imaging spacer (Sigma) with a temperature-controlled confocal microscope (Nikon C2plus, Yokohama, Japan). The cell membrane was labeled with FM4-64 (Thermofisher, Waltham, MA, USA) at 8 ng/μL. The images from bright, green (488 nm), and red (560 nm) fluorescent channels were captured for subsequential analysis. To ensure enough cell number for SEM analysis, bacteria at the late exponential phase were collected by centrifugation (Eppendorf centrifuge 5453, Hamburg, Germany) at 5000× *g* at 4 °C for 10 min, followed by two washings with M63 minimal medium, and fixed in 2.5% (*v/v*) glutaraldehyde at 4 °C. All of the procedures including dehydration, embedding, sectioning, and staining were performed according to our previous protocols [23]. The bacteria cells were then visualized using a scanning electron microscope (Hitachi S-4800, Tokyo, Japan) at an accelerating voltage of 3 kV.

### 4.5. Polymerase Chain Replication (PCR)

A DNA fragment (405 bp) from the open reading frame (ORF) region of *amiC* was amplified with the primers *amiC-Fw* (5′-ccgcaagaatagactccgca-3′) and *amiC-Rv* (5′-acgcctttaccagtcgtcag-3′) using KOD-Plus-Neo polymerase (Toyobo, Osaka, Japan). The PCR was initiated at 95 °C for 2 min followed by 30 cycles of 95 °C for 15 s, 58 °C for 15 s, 68 °C for 30 s, and a final hold of 68 °C for 10 min using SimpliAMP^TM^ Thermal Cycle (Applied Biosystems, Foster City, CA, USA). Subsequently, SYBR-Green-stained DNA bands were separated by electrophoresis (WSE-1719, ATTO, Tokyo, Japan) and visualized in the ChemiDoc™ Touch MP gel imaging system (Bio-rad, Hercules, CA, USA).

### 4.6. Genome Mutation Analysis

The *E. coli* cells grown in the M63 medium were harvested at the stationary phase for genome mutation analysis, as described previously [23]. Genome resequencing was performed by Sangon (Sangon Ltd., Shanghai, China). Genomic DNA was extracted by a Magen Bacterial DNA KF Kit (Sangon, Shanghai, China), and gDNA libraries were constructed using the NEBNext Ultra DNA Library Prep Kit for Illumina (NEB, Ipswich, MA, USA). Whole-genome re-sequencing was performed with the NovaSeq 6000 system (Illumina, San Diego, CA, USA) according to the manufacturer’s instructions. Reads were mapped to the reference sequence (NCBI accession number NC_020518.1) and subjected to the Genome Analysis Toolkit (GATK) for determining mutations, i.e., SNPs and indels. The called mutations were validated by the CleanSeq pipeline established in our previous study [57]. RAW data sets were deposited at BioProject with the accession number PRJNA924229.

## 5. Conclusions

Starting with a single clone of the *E. coli* population, we performed an FACS-directed experimental evolution aiming to select cells in a bigger size in a time-effective manner. During experimental evolution, we simultaneously analyzed the cells from each passage for dynamic morphological changes in both single-cell and population levels. Through the approach established in this study, we successfully obtained cells with a long-chain phenotype within 10 rounds of sorting-culture cycles. The whole genome sequencing revealed a stop-gain mutation in the cell division-associated gene *amiC*, leading to the dysfunction in the AmiC protein. The selection strategy based on the forward scattering here presented can be deepened in selectivity and complexity by adding multiple selection parameters, especially once imaging techniques will be fully integrated with FACS procedures [58]

## Data Availability

All data generated or analyzed during this study are included in this published article and online materials. The genome re-sequencing data is available under the NCBI BioProject with accession number PRJNA924229.

## Supplementary Materials

The following supporting information can be downloaded at: https://www.mdpi.com/article/10.3390/ijms24043243/s1.
