# Supplementary material for: Cell Sorting-Directed Selection of Bacterial Cells in Bigger Sizes Analyzed by Imaging Flow Cytometry during Experimental Evolution
Source: Int J Mol Sci. 2023 Feb 7;24(4):3243. doi: 10.3390/ijms24043243 (PMC9966196; doi:10.3390/ijms24043243)
Supplement: Supplementary file 1 [file ijms-24-03243-s001.zip › ijms-2163612-Table S1.pdf]

Table S1 Genome mutations found in the six experimental samples. The names, chromosomal position, changes in nucleotides and amino acids, and functions of genes with the detected mutations are indicated.

Samples	Gene name	Essentiality	Genome position	Change in DNA	Frequency	Change in AA	Mutant Position in AA	Domain	Pfam	Function
**MDS42 T2**	Reference genome									
**FACS-A**	*rpoB*	Essential	3612978	C→A	79.03%	Arg→Ser	p.Arg841Ser	RNA_pol_Rpb2_6(717-1264)	http://pfam.xfam.org/protein/P0A8V2	DNA-dependent RNA polymerase (RNAP) catalyzes the transcription of DNA into RNA using the four ribonucleoside triphosphates as substrates
**FACS-B**	*amiC*	No essential	2458402	C→A	99.31%	Glu→*	p.Glu382*	Amidase_3(190-405)	http://pfam.xfam.org/protein/P63883	Cell-wall hydrolase involved in septum cleavage during cell division. Can also act as powerful autolysin in the presence of murein synthesis inhibitors
**FACS-C**	No mutation found									
**Control-A**	No mutation found									
**Control-B**	*ECMDS42_RS05710*	No essential	1178583	G→C	20.64%	Val→Leu	p.Val89Leu	-		unknown
**Control-C**	*dgt*	No essential	168117	GA→G	24.05%	Ile→fs	p.Ile10fs	HD(66-182)	http://pfam.xfam.org/protein/P15723	dGTPase preferentially hydrolyzes dGTP over the other canonical NTPs
